# An Automated Approach to Improve the Quantification of Pericytes and Microglia in Whole Mouse Brain Sections

**DOI:** 10.1523/ENEURO.0177-21.2021

**Published:** 2021-11-03

**Authors:** Jo-Maree Courtney, Gary P. Morris, Elise M. Cleary, David W. Howells, Brad A. Sutherland

**Affiliations:** Tasmanian School of Medicine, College of Health and Medicine, University of Tasmania, Hobart, Tasmania, 7000, Australia

**Keywords:** brain, cell counting, image analysis, microglia, pericytes, QuPath

## Abstract

Whole slide scanning technology has enabled the generation of high-resolution images from complete tissue sections. However, commonly used analysis software is often unable to handle the large data files produced. Here, we present a method using the open-source software QuPath to detect, classify and quantify fluorescently-labeled cells (microglia and pericytes) in whole coronal brain tissue sections. Whole-brain sections from both male and female NG2DsRed x CX_3_CR1^+/GFP^ mice were analyzed. Small regions of interest were selected and manual counts were compared with counts generated from an automated approach, across a range of detection parameters. The optimal parameters for detecting cells and classifying them as microglia or pericytes in each brain region were determined and applied to annotations corresponding to the entire somatosensory and motor cortices, hippocampus, thalamus, and hypothalamus in each section. 3.74% of all detected cells were classified as pericytes; however, this proportion was significantly higher in the thalamus (6.20%) than in other regions. In contrast, microglia (4.51% of total cells) were more abundant in the cortex (5.54%). No differences were detected between male and female mice. In conclusion, QuPath offers a user-friendly solution to whole-slide image analysis which could lead to important new discoveries in both health and disease.

## Significance Statement

Quantification of cell number and distribution from whole tissue sections represents a difficult challenge in biomedical research. Slide scanning microscopes generate high-resolution images of complete tissue sections but most common image analysis software packages struggle to cope with the large data files they produce. We provide a method for quantifying pericyte and microglia cell numbers in whole-brain tissue sections using QuPath, an open-source software designed specifically to overcome this challenging roadblock.

## Introduction

The mammalian brain is a large and complex organ with numerous cell types. The parenchymal cells of the brain, including neurons, microglia, astrocytes, oligodendrocytes, and oligodendrocyte precursor cells (OPCs), coexist alongside cells lining the walls of the ventricles (ependymal cells), and cells forming the blood vessels of the brain (endothelial cells, pericytes, and vascular smooth muscle cells). Accurately determining the density and spatial relationships between these different cell types, in any given brain region, can provide clues to the importance and functions of each cell type in both health and disease.

Brain cells can be visualized by different forms of microscopy, including brightfield or fluorescence, following histologic or immunohistochemical processing of isolated cells or tissue sections. The quantitative and spatial analysis of cells has traditionally been limited by the field of view of the microscope and the workload associated with analysis of multiple fields of view, which can hinder the detection of patterns across larger regions. Virtual microscopy technology, which enables the scanning of whole microscope slides at high resolution, has emerged in the last two decades to overcome these limitations (Al-Janabi et al., 2012). Although whole slide scanning has predominantly been adopted in the field of diagnostic pathology, basic research laboratories can also benefit from the analysis of different cell types over large areas of tissue.

Whole slide scanning has, however, created a new roadblock. The files generated by slide scanning microscopes are large and difficult to handle for many common image analysis programs, including open-source software such as ImageJ and CellProfiler. To overcome this, researchers resort to reducing image resolution (which can reduce the accuracy of the analysis), sampling smaller regions of interest (as a representative of the larger whole), or painstakingly analyzing the whole by consecutively imaging and analyzing small regions of interest. Recently, the open-source application QuPath was specifically developed to better enable pathologists and researchers to analyze whole slide images (Bankhead et al., 2017). QuPath can integrate with ImageJ and other packages to reuse carefully developed analysis tools and allows the user to rapidly analyze high resolution images without requiring expensive, specialized computing facilities and without having to rely on sampling smaller regions of interest. The initial application for QuPath was for tumor identification and biomarker evaluation in cancer (Bankhead et al., 2018; Humphries et al., 2018; Ledys et al., 2018; Loughrey et al., 2018), but its extensible platform provides the flexibility to analyze large and complex images across a range of biomedical settings (Bankhead et al., 2017). For example, the effective identification of GFAP-positive astrocytes across whole-brain sections recently provided a demonstration of the use of QuPath in neurologic microanatomy (Finney et al., 2021). Here, we demonstrate the potential of QuPath to detect fluorescently-labeled brain cells, in particular microglia and pericytes.

Both microglia and pericytes are distributed widely throughout the brain and have important functions in health and disease. Microglia, historically considered the innate immune cells of the brain (Morris et al., 2013), are unique to the CNS, with key roles in sculpting, maintaining and modifying neural circuitry through their influence on synaptic and structural plasticity (Colonna and Butovsky, 2017). Pericytes, a cell present throughout the CNS and the periphery, have numerous roles in the brain including the regulation of cerebral blood flow (Hall et al., 2014) and the maintenance of the blood-brain barrier (Armulik et al., 2010). The historical and contemporary research on both cell types in health and disease have been extensively reviewed elsewhere (Morris et al., 2013; Sweeney et al., 2016; Colonna and Butovsky, 2017; Brown et al., 2019; Beard et al., 2020).

In this study, we have used QuPath to quantify the relative numbers of microglia and pericytes in whole coronal brain tissue sections derived from transgenic mice expressing fluorescently-labeled microglia and pericytes. We describe, for the first time, the use of QuPath to analyze images of whole mouse brain sections for fluorescently-labeled cells in an automated fashion. We also highlight optimization processes that permit quantification of microglia and pericytes under different imaging circumstances in different brain regions. Our approach can be easily applied to any brain region or other tissue types, or other fluorescently-labeled cells, and can be used to quantify cell numbers in different disease states. This approach further enhances the capabilities of QuPath to analyze whole-brain section fluorescence in an automated fashion using evidence-based parameter selection.

## Materials and Methods

### Animals, tissue acquisition, and processing

All animal procedures were approved by the Animal Ethics Committee, University of Tasmania (A0018608) and conformed with the Australian National Health and Medical Research Council (NHMRC) Code of Practice for the Care and Use of Animals for Scientific Purposes, 2013 (eighth edition). Hemizygote NG2DsRed transgenic mice (The Jackson Laboratory stock #008241) were backcrossed onto a C57BL/6J background and crossbred with CX_3_CR1^GFP/GFP^ transgenic mice (The Jackson Laboratory stock #005582, C57BL/6J background) to produce NG2DsRed x CX_3_CR1^+/GFP^ mice. Mice were group housed in Optimouse caging on a 12/12 h light/dark cycle (lights on: 7 A.M. to 7 P.M.) with *ad libitum* access to standard chow and water.

Eight 12-week-old male and female NG2DsRed x CX_3_CR1^+/GFP^ mice weighing 18.7–30.3 g (Table 1) were killed with a lethal intraperitoneal injection of pentobarbitone (300 mg/kg) and immediately transcardially perfused with 4% paraformaldehyde (PFA; pH 7.4). Whole brains were harvested and postfixed in 4% PFA for 1.5 h, then transferred to 30% sucrose in 1× PBS until they sank. Whole brains were embedded in Cryomatrix embedding resin (Shandon, catalog #6769006) and frozen at −80°C until cryosectioning. 40 μm coronal sections were cut at −18°C using a cryostat and placed free floating in 1× PBS. Using the Allen Brain Atlas as a guide (Lein et al., 2007), tissue sections −1.70 mm from bregma were mounted onto microscope slides (Dako, catalog #K802021-2), allowed to dry upright for 30 min, washed for 5 min in 1× PBS Tween (0.1%), rinsed for 30 s in 1× PBS followed by 10 s in distilled H_2_O, air dried for 5 min, then coverslipped with Prolong Gold antifade reagent with DAPI (Life Technologies, catalog #P36935).

**Table 1:** Descriptive statistics of animals and tissue

	Male (*n* = 3)	Female (*n* = 5)	*p* value
Weight (g)	27.77 ± 2.37	19.74 ± 1.05	*p* = 0.0005 ***
Age (d) ^#^	87.67 ± 2.52	85.00 ± 0.00	*p* = 0.2079 ns
Tissue slice area (mm^2^)	42.17 ± 0.86	42.03 ± 1.25	*p* = 0.8739 ns

All statistics are mean ± SD. Male and female groups were compared with an unpaired *t* test (# with a Welch’s correction when variances were inhomogeneous between groups); ***p < 0.001; ns, not significant.

### Image acquisition

Images were acquired using a VS120 Virtual Slide System (Olympus). Whole slides were first scanned in the DAPI channel (Excitation (Ex): 388 nm; Emission (Em): 448 nm) at 2× magnification and then the outlines of whole coronal tissue sections were traced for scanning at 40× magnification. A focus-map (the highest density possible) was auto-generated across the entirety of each coronal section and the plane of focus was automatically determined based on the DAPI channel. DAPI (Ex: 388 nm; Em: 448 nm), DsRed (Ex: 576 nm; Em: 625 nm), and GFP (Ex: 494 nm; Em: 530 nm) signals were imaged in the same focal plane. Optimum exposure times were initially determined manually and then kept consistent for all images (DAPI, 50 ms; DsRed, 100 ms; GFP, 50 ms). The .vsi files generated were approximately 2 GB each in size.

### Image analysis: computing and software

All image analysis was performed on a standard desktop computer with an Intel Core i7-6700 processor and 16-GB installed memory running Windows 10 and QuPath-0.2.3. Script development was aided by IntelliJ IDEA 2020.3.2 (Community edition). Analysis of exported data were performed using Microsoft Excel and GraphPad Prism 9.0.2.

**Figure 1.**
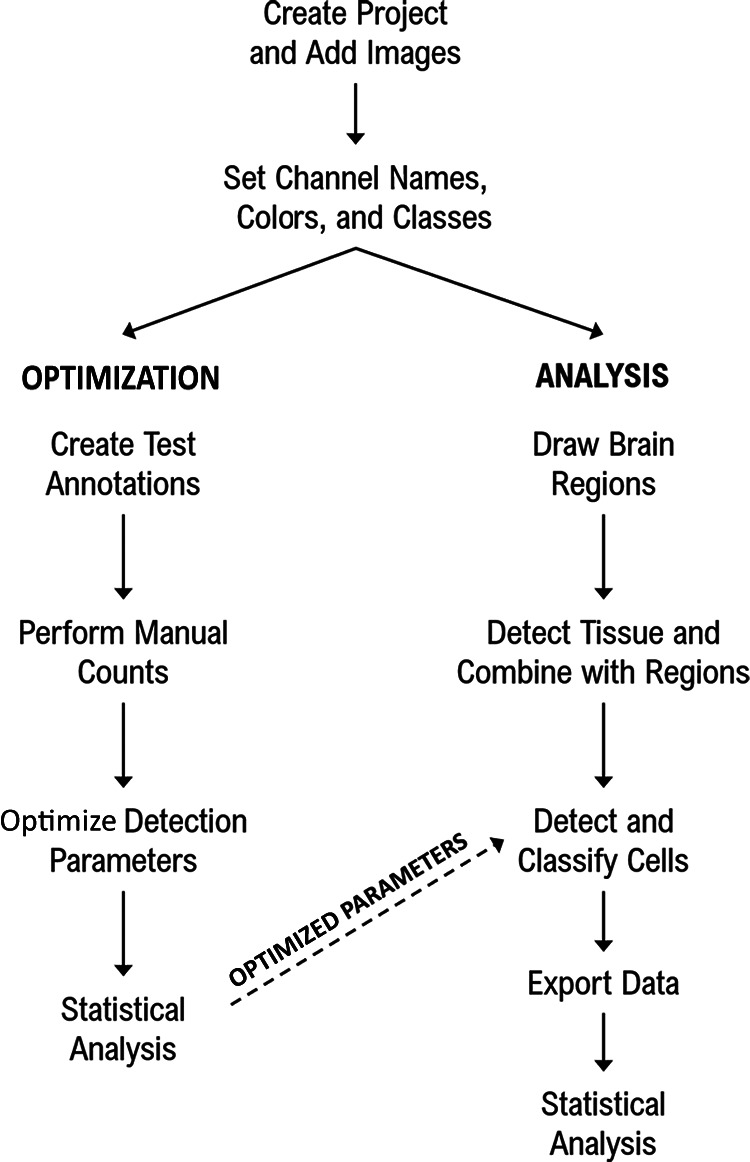
Analysis pipeline. Flowchart summarising the steps taken to optimize analysis parameters and then to detect and classify fluorescently labeled cells in whole mouse brain sections in QuPath.

The method described here was based on the Multiplexed Analysis Tutorial found in the QuPath online documentation (Bankhead, 2020) with adaptations made for whole-brain section analysis and the specifics of the tissue used here. An overview of the analysis pipeline is shown in Figure 1.

### Code accessibility

Source code for the scripts and classifiers used here is freely available online at https://github.com/jo-maree/qupath-scripts-2021. The groovy and json scripts (text files) are available as Extended Data 1.

10.1523/ENEURO.0177-21.2021.ed1 Extended Data 1 Scripts and classifiers. Zip file containing the scripts (.groovy files) and classifiers (.json files) that were developed for use in QuPath in this study. Download Extended Data 1, ZIP file.

### Image analysis: project setup

A QuPath project was created to allow the application of scripts and classifiers across multiple images. All .vsi files were loaded with the image type set to “fluorescence”. QuPath does not hold the actual image files but rather links to the original images, and so it was ensured that the project file and original image files were never separated. The project was duplicated to create separate projects for optimization and postoptimization analysis.

### Image analysis: channel names, colors, and classes

**Figure 2.**
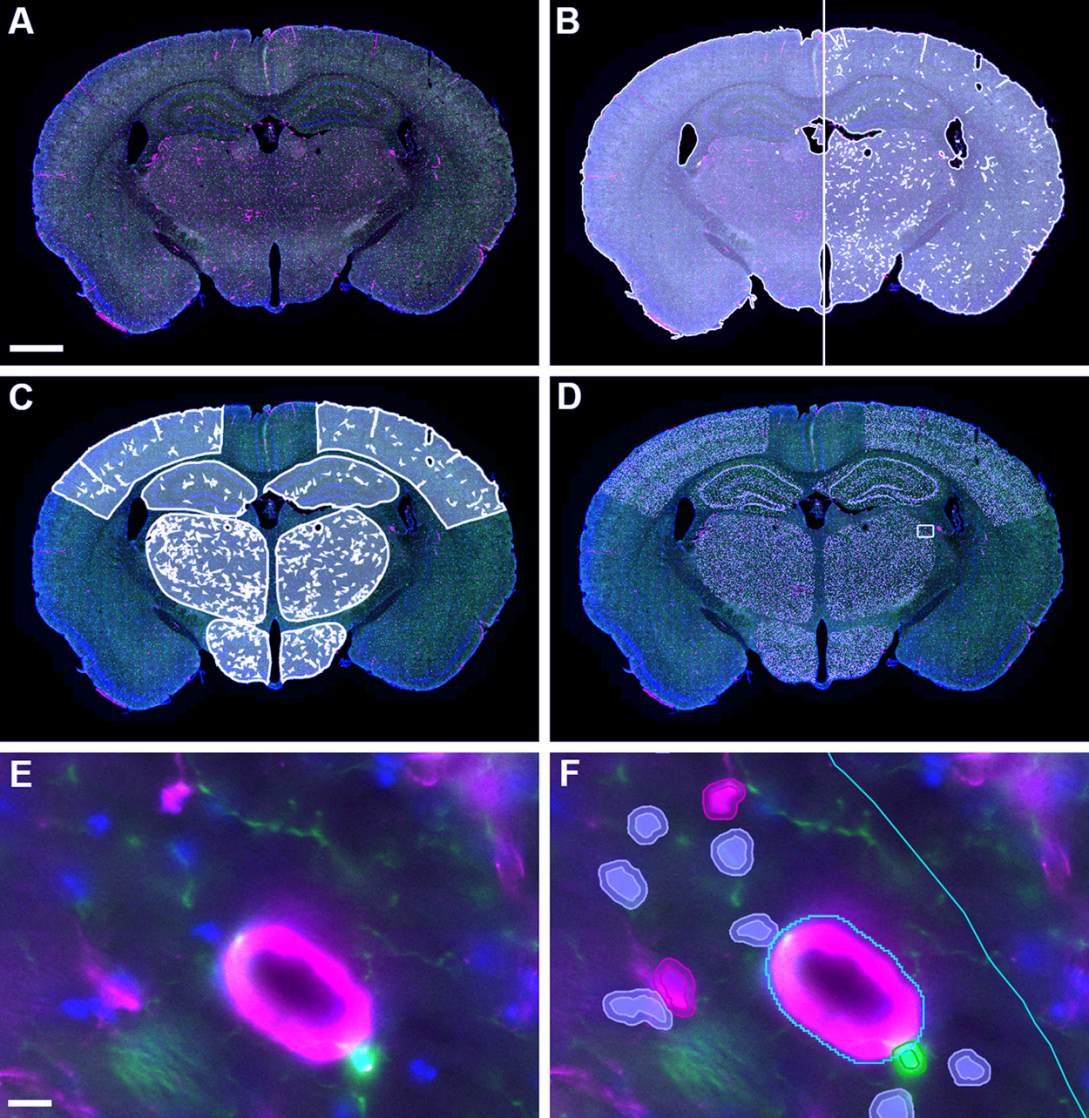
Stages of QuPath analysis on whole mouse brain sections. ***A***, Imported image following correction of channel colors. ***B***, Initial tissue detection (left) and following subtraction of large vessels and edges (right). ***C***, Brain regions intersected with detected tissue. ***D***, Overlay of detected nuclei (gray) on tissue. ***E***, High-magnification view of a region of the thalamus (indicated by box in ***D***). ***F***, With annotation boundaries in cyan and detected nuclei outlined in magenta (DsRed), green (GFP), and gray (other). Scale bars: 5 mm (***A–D***) and 10 μm (***E–F***). DAPI, GFP, and DsRed signal are colored blue, green, and magenta, respectively. Extended Data Figure 2-1 shows an example of the placing of annotations for manual cell counting.

Appropriate channel colors and names (DAPI, DsRed, GFP) were set for all images as a batch using the script “Channels and colours.groovy” and classes were created from these channel names using the “populate from image channels” command (Fig. 2*A*). In our figures the DAPI, DsRed, and GFP have been pseudo colored blue, magenta, and green, respectively.

10.1523/ENEURO.0177-21.2021.f2-1 Extended Data Figure 2-1 Example of optimization annotations. We placed 300 × 200-μm regions of interest in the upper cortex (layers 1–3), lower cortex (layers 4–6), hippocampus (including dentate gyrus), hippocampus (including CA1/CA3 boundary), thalamus, and hypothalamus of each brain section for the purposes of manually counting cells. Scale bar: 800 μm. Download Figure 2-1, TIF file.

### Optimization: selection of test annotations and manual counting

QuPath has the functionality to analyze entire brain sections or smaller regions of interest, and different regions of the brain may require different parameters for optimal cell detection. To determine the optimal parameters for our DAPI-positive nuclei detection and the GFP-positive/DsRed-positive cell classifications across the brain, for each image (*n* = 8) a small annotation (300 × 200 μm) was drawn in each of six brain regions of interest: upper cortex (layers 1–3), lower cortex (layers 4–6), hippocampus (including dentate gyrus), hippocampus (including CA1/CA3 boundary), thalamus, and hypothalamus (Extended Data Fig. 2-1). In each annotation area, the total number of cells (DAPI-stained nuclei) and the number of DsRed-positive and GFP-positive cells were counted manually by an experienced researcher (G.P.M.) using the points annotation function of QuPath.

### Optimization of cell detection parameters

In QuPath, fluorescent cell detection can be performed using any channel but most commonly utilises a nuclear stain such as DAPI to first detect all cells. The in-built Cell Detection algorithm requires the selection of various parameters. It is possible to rigorously optimize each of these parameters (i.e., pixel size, background radius, median radius, sigma, minimum area, maximum area, and threshold) individually, or by mixing and matching different settings for each, then comparing these settings to manual counts to ensure accurate automated detection of cells. Manually changing these parameters to test each possible combination is time consuming, so we designed a script capable of doing this automatically (“Optimisation of cell detection.groovy”).

To illustrate the importance of optimizing cell detection parameters we present our optimization of one key detection parameter: the DAPI intensity threshold. For simplicity, the other cell detection parameters were kept to QuPath’s defaults, except for sigma = 1.5 and cell expansion = 2 μm (a cell expansion allows for the detection of fluorescent labeling outside of the nucleus).

For each test annotation, DAPI intensity thresholds were tested in increments of 25, beginning at 50, and ending at 1000. The number of detected cells at each threshold was compared with manual counts of DAPI-positive cells through the calculation of % difference using the equation below:

$$\%Difference=\left(\frac{Ac-Mc}{Mc}\right)\times 100.$$

A % difference of 0 indicated that both automated (Ac) and manual (Mc) counts were equal, >0 indicated that automated counts were higher than manual counts, <0 indicated that automated counts were lower than manual counts. These values were used to determine the optimal threshold for each region of the brain.

### Optimization of fluorescent intensity thresholds for cell classification

After optimizing the cell detection parameters, we optimized the intensity threshold parameters for classifying detected cells as DsRed-positive or GFP-positive. The inbuilt positive cell detection plugin was applied to the same small annotations used for optimizing DAPI cell detection. Here, the DAPI threshold for nuclear detection was set to the previously determined optimum for each specific brain region while the threshold (measuring the mean value in the cell) was tested using a script (“Optimisation of cell classification.groovy”): first for DsRed (thresholds between 200 and 550 in increments of 25) and then for GFP (thresholds between 100 and 450 in increments of 25).

As with DAPI detection, the number of DsRed-positive and GFP-positive cells detected at each threshold was compared with the manual counts (with visual verification) to determine the optimal DsRed and GFP thresholds for each brain region.

### Image analysis: annotation of brain regions

For each image in the analysis project, the brush tool was used to draw annotations for the somatosensory and motor cortex (hereon referred to as ‘cortex’), hippocampus, thalamus and hypothalamus in both left and right hemispheres using the Allen Mouse Brain Atlas as a guide (Lein et al., 2007). The selected regions in the left and right hemispheres were merged to form a single annotation for each region and each annotation was named appropriately using the “set properties” dialog box. Two scripts were used to assist this process: “Save Annotations.groovy” exports the annotations for the first image to a file which can then be imported back into the remaining images with “Import Annotations.groovy”. Annotations for each image were individually adjusted using the brush tool to fit the specific anatomy of each section.

### Image analysis: tissue and vessel detection

For each tissue section, the tissue area was defined using a pixel classifier based on the average value of the three channels at high resolution with a Gaussian prefilter, smoothing sigma = 2.0 and threshold = 50 and an annotation created with a minimum area of 1,000,000 μm^2^ and a minimum hole size of 1000 μm^2^. This annotation was eroded by 40 μm (using “expand annotations” set to −40 μm) to reduce the effects of tissue processing artefacts around the edge of the tissue, and fragments <10,000 μm^2^ were removed (using “remove fragments and holes”). In order to exclude large DsRed-positive vessels, likely reflecting DsRed-positive vascular smooth muscle cells, rather than capillary pericytes, a second pixel classifier was created using the DsRed channel at high resolution with a Gaussian prefilter, smoothing sigma = 2.0 and threshold = 400. An annotation was created from this classifier with a minimum size of 150 μm^2^ and a minimum hole size of 1000 μm^2^. Next, the “Vessels” annotation was subtracted from the “Tissue” annotation. A script was created to incorporate the pixel classifiers and automate these steps (“Tissue Dectection.groovy"”) and run as a batch for the project (Fig. 2*B*). Finally, the intersection between the detected tissue (minus large vessels) and the predefined brain region annotations was calculated using the script “Intersect ROIs.groovy” (Fig. 2*C*).

### Image analysis: detection and classification of cells

To accommodate the need for different color thresholds in different brain regions, a specific composite object classifier was created for each brain region and saved in the QuPath project’s “object_classifiers” folder.

**Figure 3.**
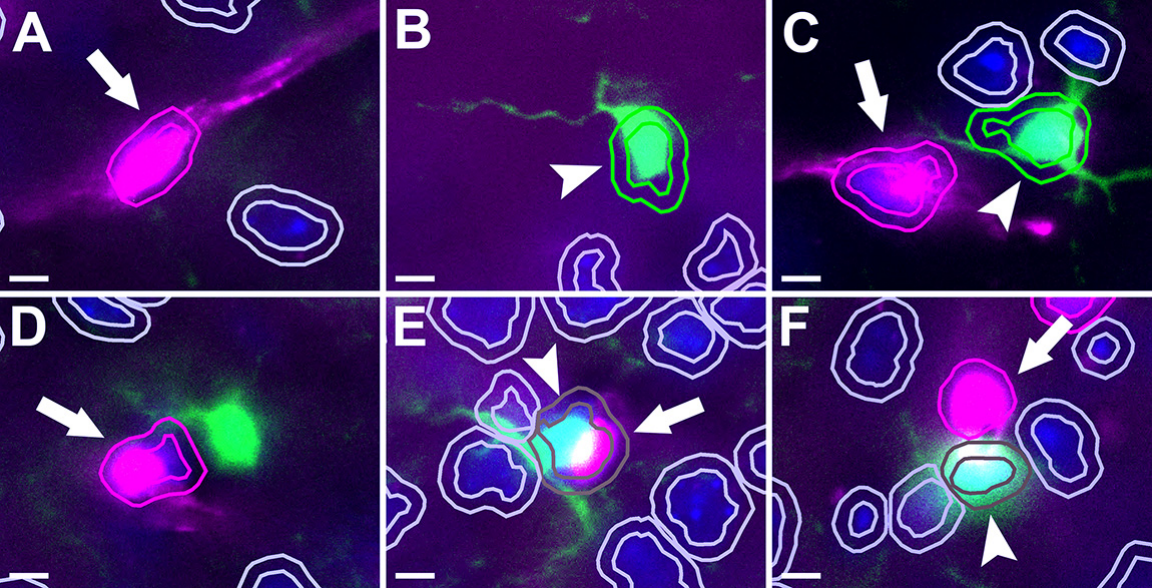
Examples of detected cells. DsRed-positive pericytes are indicated with arrows, GFP-positive microglia with arrowheads. Each cell detected using DAPI staining is shown as an inner ring (nucleus) and outer ring (2 μm expansion) colored according to classification (magenta, DsRed-positive; green, GFP-positive; brown, DsRed-positive and GFP-positive; gray, DsRed-negative and GFP-negative). ***A–C***, Appropriately classified cells. ***D***, The pericyte is classified appropriately, but the microglia is not detected because of the nucleus being out of the plane of the section. ***E***, A microglia and pericyte that are in close contact and were not able to be separated by the nuclear detection leading to a dual classification. ***F***, The pericyte is appropriately classified, but some DsRed fluorescence has overlapped with a microglia to cause a dual classification. This figure illustrates two of the possible reasons for cells to be dual-classified; however, overall occurrence of dual-classified cells is low (see Fig. 5*B*). Scale bars: 5 μm.

Finally, cell detection and classification was combined into a single script to run for the whole project (“Cell Detection and Classification.groovy”), using the predetermined DAPI, DsRed, and GFP thresholds for each region (Fig. 2*D*). Examples of cell detections are shown in Figures 2*E*,*F*, and 3.

### Image analysis: export of measurements

Annotation measurements, including area and number of detections, were exported for each brain region using QuPath’s measurement exporter.

### Statistical analysis

All statistical analyses were performed using GraphPad Prism 9.0.2. The parametric tests outlined below were only undertaken if data passed normality assessment using the Shapiro–Wilk test. Where data did not pass normality, a ROUT test to detect outliers was conducted, and where an outlier was detected, this datapoint and linked datapoints were removed. Whole animal and tissue slice measurements were compared with unpaired *t* tests with a Welch’s correction was used if variances were inhomogeneous (see Table 1). The correlation of manual counts compared with automated counts was performed using the Pearson’s correlation coefficient. Cell counts were compared between brain regions by repeated measures one-way ANOVA with *post hoc* Tukey’s multiple comparison tests. For the repeated measures ANOVA, the Geisser–Greenhouse correction was applied to account for variation in sphericity. Sex effects on cell counts were tested with repeated measures two-way ANOVA. A *p* < 0.05 was considered statistically significant.

## Results

### Animal and tissue characteristics

Brain slices for analysis were taken from three male and five female mice. While male mice were significantly heavier than the female mice, there was no difference in the area of the tissue slices analyzed (Table 1).

### Optimization

**Figure 4.**
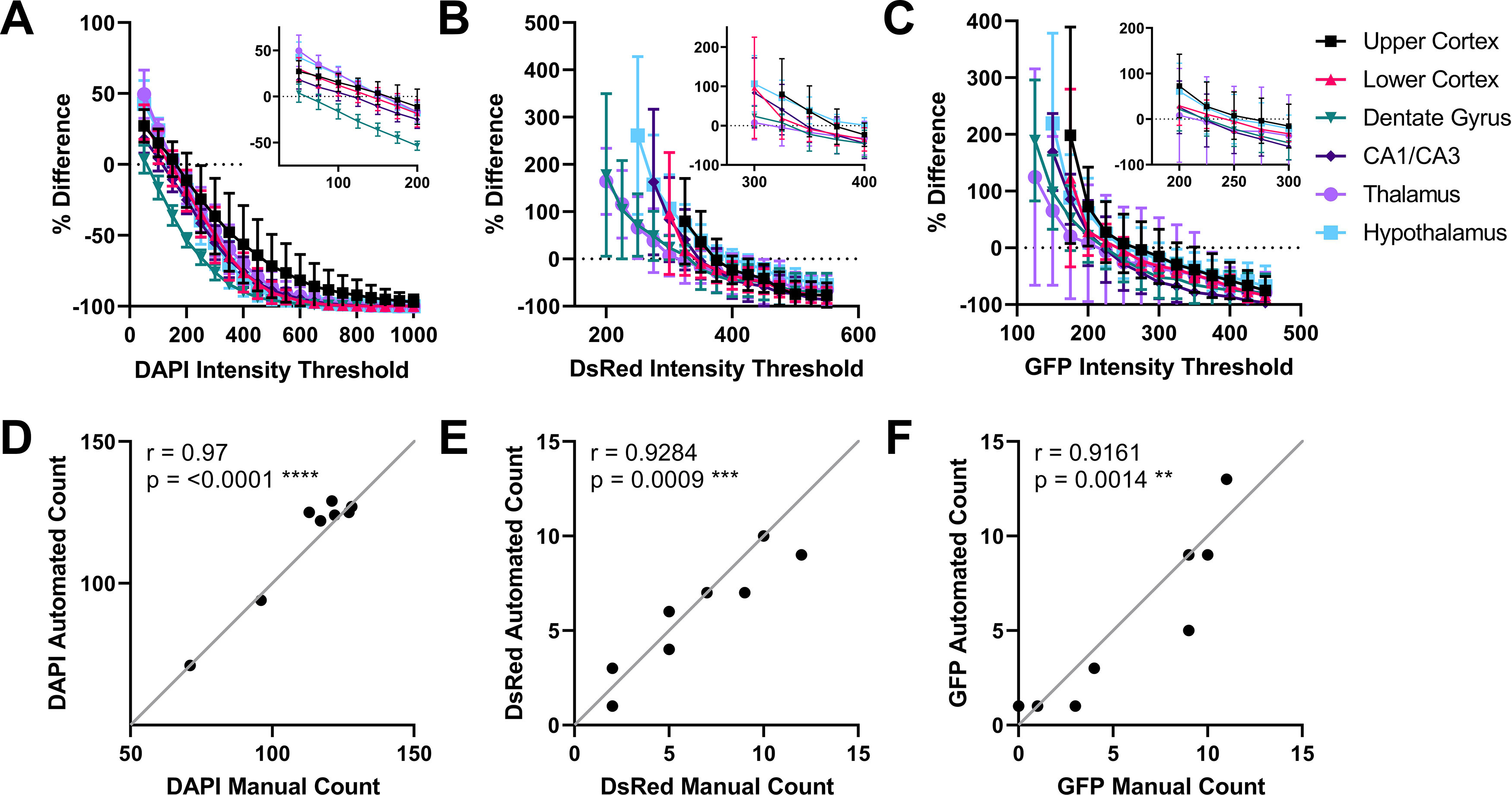
Optimization of cell detection and classification thresholds. Counts generated by QuPath’s cell detection/positive cell detection algorithm were compared with manual cell counts to generate a % difference (dotted line at 0%) with a range of intensity thresholds across six brain regions for (***A***) DAPI, (***B***) DsRed, and (***C***) GFP (*n* = 8, mean ± SD). Insets show more detail at thresholds where the percentage difference crosses zero. For DsRed and GFP, data for thresholds with SDs over 200 have been excluded from the graphs to more clearly visualize the optimum threshold for each brain region. Extended Data Figure 4-1 shows intensity threshold analyses for annotations of individual brain regions. Example correlations of automated counts to manual counts for the thalamus using the final optimized values for (***D***) DAPI, (***E***) DsRed, and (***F***) GFP. Extended Data Figure 4-2 includes correlation charts for all optimized brain regions. Correlations between automated and manual counts were calculated using the Pearson’s correlation coefficient (*r*). **p < 0.01, ***p < 0.001, ****p < 0.0001.

After running the QuPath Cell Detection algorithm through our custom script, we compared manual cell counts from 300 × 200 μm annotations in six different regions to the number of DAPI-positive nuclei detected by the algorithm across multiple different DAPI intensity thresholds (summarized in Fig. 4*A*; for individual comparisons, see Extended Data Fig. 4-1). Optimal DAPI-thresholds (Extended Data Fig. 4-1, arrows) were selected as the thresholds which provided the greatest accuracy (i.e., closest mean to manual counts) and least variability (i.e., smallest SD), with a preference for undercounting (false negatives) rather than overcounting (false positives). For the cortex, thalamus and hypothalamus, the optimal threshold was determined to be 150, while the hippocampal regions required lower thresholds between 50 and 100 for accurate cell detection. For the hippocampus, we ideally required a threshold that would be applicable to the whole region (i.e., including CA1/CA3 and DG in the same analysis). We therefore chose a threshold of 75 for the entire hippocampus, which provided accurate counts in both regions. For each brain region, the number of cells detected using the optimized DAPI thresholds significantly correlated to number of cells counted manually (Fig. 4*D*; Extended Data Fig. 4-2).

10.1523/ENEURO.0177-21.2021.f4-1 Extended Data Figure 4-1 Detailed optimization of cell detection and classification thresholds. Counts generated by QuPath’s cell detection/positive cell detection algorithm were compared to manual cell counts to generate a % difference (dotted line at 0%) with a range of intensity thresholds across six brain regions for DAPI, DsRed, GFP (*n* = 8, mean ± SD). Data for lower thresholds with large SDs have been excluded from the graphs in order to clearly visualize the optimum threshold for each region and channel (indicated with an arrow). Download Figure 4-1, TIF file.

10.1523/ENEURO.0177-21.2021.f4-2 Extended Data Figure 4-2 Correlation of manual counts to automated counts at final optimized thresholds. For each optimized threshold, the Pearson’s correlation coefficient (*r*) between cells counted manually and automated counts by QuPath was calculated. Download Figure 4-2, TIF file.

Next, we used another custom script to iteratively apply QuPath’s positive cell detection algorithm, using the DAPI thresholds optimized for each brain region, to test multiple DsRed and GFP intensity thresholds. As with the optimization of DAPI thresholds, we compared these to manual counts to determine the optimal thresholds for DsRed-positive and GFP-positive cell detection in each region (summarized in Fig. 4*B*,*C*; for individual comparisons, see Extended Data Fig. 4-1). Again, thresholds (Extended Data Fig. 4-1, arrows) were selected for accuracy, low variability, and with a preference for false negatives. The number of cells detected using the optimized DsRed and GFP thresholds correlated to the number of DsRed-positive and GFP-positive cells counted manually (Fig. 4*E*,*F*; Extended Data Fig. 4-2).

The final optimized thresholds for each channel and region are listed in Table 2. Note, these optimized thresholds are only applicable to our specific tissue and would be expected to vary in each laboratory based on the tissue processing methodology and imaging parameters.

**Table 2:** Optimized intensity thresholds for cell detection and classification by brain region

	DAPI	DsRed	GFP
Cortex	150	375	250
Hippocampus	75	350	225
Thalamus	150	325	200
Hypothalamus	150	400	250

### Cell detection and quantification in different brain regions

**Figure 5.**
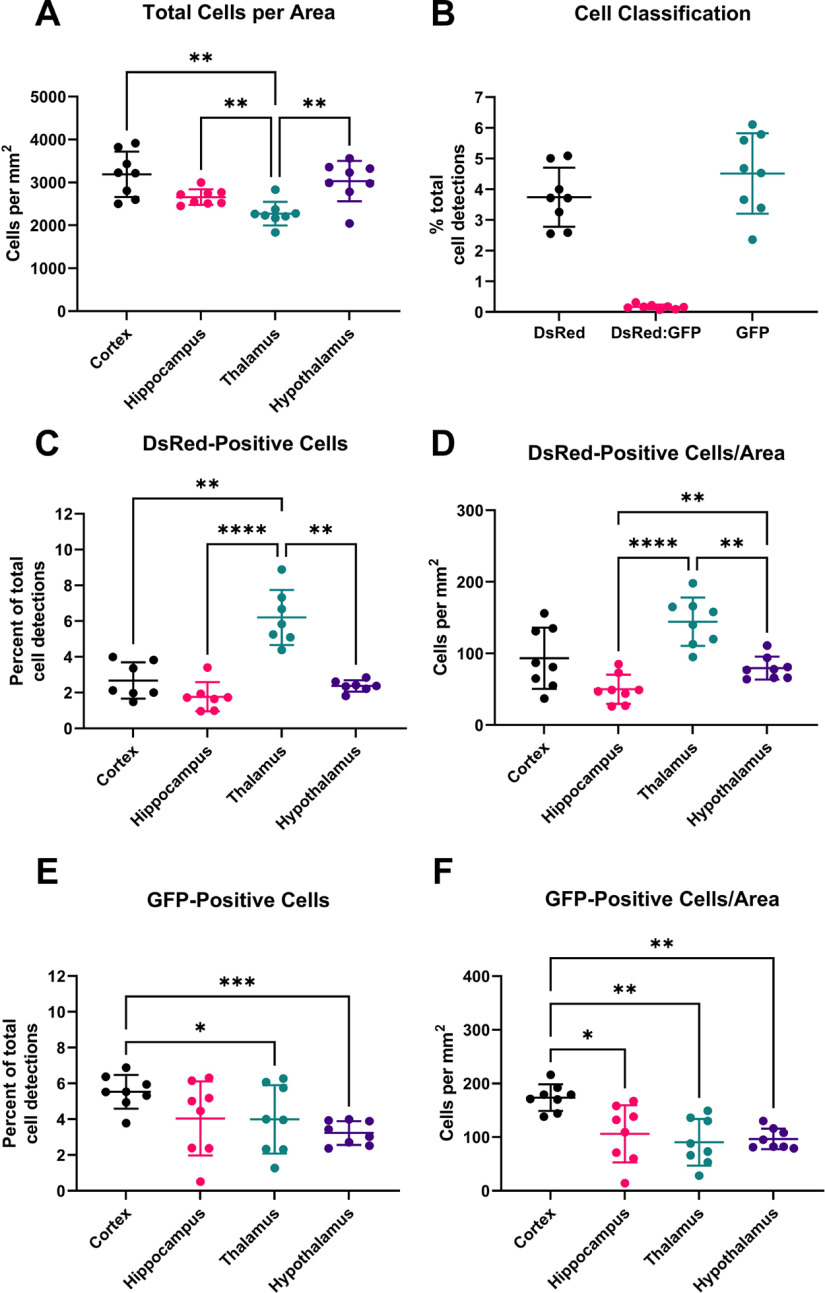
Detection and classification of cells. ***A***, Total cells detected per mm^2^ tissue area in each brain region by DAPI nuclei staining. ***B***, Percentage of cells by classification across all brain regions measured. DsRed-positive cells by brain region (***C***) as percentage of total cell detections and (***D***) per mm^2^. GFP-positive cells by brain region (***E***) as percentage of total cell detections and (***F***) per mm^2^. Statistical analysis by repeated measures one-way ANOVA with *post hoc* Tukey’s multiple comparison test. All data passed the Shapiro–Wilk test for normality except hypothalamus in ***C***. An outlier was identified and removed from this group and data from the same brain slice was removed across all brain regions, which subsequently passed normality. All data underwent the Geisser–Greenhouse correction to account for variation in sphericity; *p < 0.05, **p < 0.01, ***p < 0.001, ****p < 0.0001. Extended Data Figure 5-1 shows detection and classification analyses comparing male and female mice with no statistically significant differences observed.

Following the optimization of cell detection parameters on small annotations, we applied our cell detection script with specific object classifiers to each region of interest within our coronal sections (one section per animal, *n* = 8). Automated cell detection (based on DAPI staining) was performed on each region of interest in each brain section (Fig. 5*A*). The total number of cells per area differs significantly between brain regions (one-way ANOVA, *p *=* *0.004).

10.1523/ENEURO.0177-21.2021.f5-1 Extended Data Figure 5-1 Detection and classification of cells by sex. Total cells (***A***), DsRed-positive cells (***B***), and GFP-positive cells (***C***) detected per mm^2^ tissue area. No effect of sex was found by two-way ANOVA. Download Figure 5-1, TIF file.

Across all regions tested, 3.74% (±0.90%) detected cells were classified as DsRed-positive (pericytes), 4.51% (±1.23%) were classified as GFP-positive (microglia), and 0.17% (±0.07%) were classed as positive for both DsRed and GFP (Fig. 5*B*). One reason for the classification of a subset of cells as positive for both markers is the close proximity of some pericytes to microglia, making it difficult for the automated analysis to distinguish individual cells that have overlapping DsRed and GFP fluorescence (Fig. 3*E*,*F*). Given the small numbers, these cells were excluded from further analysis.

The proportion of total DAPI-positive cells that were identified as DsRed-positive or GFP-positive differed significantly between brain regions. The thalamus had a significantly higher proportion of DsRed-positive pericytes (6.20 ± 1.54%) compared with the other brain regions assessed (cortex: 2.68 ± 1.02%, *p* = 0.0062; hippocampus: 1.77 ± 0.81%, *p* <0.0001; and hypothalamus: 2.37 ± 0.32%, *p* = 0.0014; Fig. 5*C*). The cortex had a significantly higher proportion of GFP-positive microglia (5.54 ± 0.94%) than other brain regions assessed (hippocampus: 4.05 ± 2.07%, *p* = 0.0597; thalamus: 3.99 ± 1.91%, *p* = 0.0311; and hypothalamus: 3.23 ± 0.66%, *p* = 0.0001; Fig. 5*E*). A similar pattern of regional differences was evident when cells counts were expressed as cells/mm^2^ of tissue area (Fig. 5*D*,*F*).

No statistical differences were identified between male and female mice for any of the cell detection or classification measures described (Extended Data Fig. 5-1).

## Discussion

In this study, we have detailed an automated method to identify fluorescently labeled nuclei, microglia and pericytes in high-resolution images using QuPath. To validate our approach we compared automated nuclei, pericyte and microglia counts to manual counts. The approach we describe offers an unbiased, replicable method to quantitate nuclei and cell numbers across large fluorescently labeled tissue sections, drastically reducing the time taken to obtain cell counts. Below we discuss the importance of optimizing the QuPath cell detection parameters for each project. Furthermore, we highlight limitations in our QuPath methodology and we discuss other useful features of QuPath beyond those we have assessed in this work.

QuPath represents a significant advance in biomedical image interpretation by enabling batch analysis of large (>2 GB), pyramidal image files produced by slide scanners in a scriptable, open-source environment on a standard desktop computer, without the need to downsample or limit analysis to small regions of interest. Since its release in 2017, over 700 publications have used QuPath, the vast majority of which have analyzed tissue sections stained with chromogenic immunohistochemistry. Previous publications have described the use of QuPath to assess the staining intensity or cell number of specific cell types within the brain including astrocytes (Finney et al., 2021), microglia (Bevan et al., 2018; Morriss et al., 2020), and neurons (van Olst et al., 2021). These studies utilized chromogenic immunohistochemistry and so could not perform analyses of multiple cell types on single tissue sections. QuPath also has the capability to analyze tissue labeled with fluorescent markers, a feature which offers a number of advantages including higher dynamic range and easier multiplexing, leading to better identification of colocalized targets and allowing the user the ability to determine spatial relationships between different cell types. It also enables quantification of cells expressing genetically-encoded fluorescent proteins, which can offer advantages over fluorescent immunohistochemistry. Here, we provide the first investigation utilizing QuPath to detect multiple types of fluorescently-labeled cells in mouse brain tissue sections, specifically pericytes expressing DsRed and microglia expressing GFP. In addition, we provide a series of scripts that automate the process of optimizing crucial detection parameters in a systematic, transparent, and unbiased way.

When using automated approaches for cell detection and quantification it is prudent to optimize the automated cell detection parameters for each individual project because of differences in staining protocols, image acquisition and regional differences in staining intensity in subregions of a tissue sample (Roeder et al., 2012). In QuPath, detection parameters may be optimized empirically by adjusting individual parameters until the detected cells match those observed by the researcher, or can be determined using a more systematic approach, as we have employed in this study. As a proof of principle, we undertook a detailed optimization of the fluorescence intensity thresholds required for accurate detection of cells in all three channels. To expedite this process, we designed a custom script to enable rapid testing of multiple fluorescent intensity thresholds, without having to manually alter them. Although the fluorescent signal we analyzed was provided by genetically-encoded fluorescent proteins providing a relatively clean signal, immunofluorescence techniques with higher levels of background staining are also frequently utilized for cell detection. Therefore, we designed the script to also test other cell detection parameters available in QuPath, for example sigma and background radius (a rolling-ball background reduction measure), providing the user the ability to automate the process of defining the optimal parameters for the analysis of their tissue of interest. This provides flexibility and advances the capabilities of producing accurate assessment of cells in fluorescently-labeled whole-brain sections.

The data we obtained using this approach (Fig. 4) enabled us to determine that the fluorescent intensity thresholds for cell detection were different in the various subregions we analyzed in our coronal mouse brain slices. This conclusion was reached by comparing the automated counts at different thresholds to manual counts, with the assumption that our manual counts represent ground truth. Interestingly, the curves produced by changing fluorescence intensity thresholds (Fig. 4; Extended Data Fig. 4-1) provide a useful insight into the importance of careful optimization. This is particularly important when classifying cells that represent a low proportion of the total cells present in a region. The successful detection of DAPI-labeled nuclei was relatively insensitive to changes in threshold intensity in the cell detection parameters and thus variability was consistent across the intensity thresholds we tested. This is partly because of the number of cells counted (50–150 per region of interest) and the fact that thresholding is one of many factors contributing to cell detection within the algorithm. However, when changing the threshold for the classification of detected cells as either DsRed or GFP-positive, the small number of actual positive cells (<15 per region of interest) and the fact that intensity threshold is the only classification parameter used led to higher variability at lower thresholds and convergence to zero (represented by −100% difference in Fig. 4*A–C*) at higher thresholds.

We have not yet determined why different subregions required different intensity thresholds for accurate cell detection in our coronal mouse brain sections. This could be a biological feature of the tissue. For instance, microglia in the thalamus may express a different level of CX_3_CR1-GFP than cells in the cortex, therefore requiring a different intensity threshold for accurate quantification. Alternatively, it could be a technical artifact. For example, the edges of the tissue often have higher background fluorescence than regions in the middle of the tissue, thereby requiring a higher intensity threshold to avoid false positives. Whatever the cause, the finding that different subregions of interest required different cell detection parameters highlights the importance of optimizing detection parameters in each experiment, especially when attempting to compare cell numbers from region to region.

The classification of cells with expression of two separate fluorescent proteins in a single section raises the possibility some cells may be “dual-classified,” that is, a single cell may be detected as expressing both markers (Fig. 3*E*,*F*). Whether these are truly cells expressing both markers, or whether this is merely an artifact of the imaging and analysis process, will depend on the markers in question. In our study we did not expect DsRed and GFP to colocalize as NG2 and CX_3_CR1, considered markers of pericytes and microglia in the brain, respectively, have not, to the best of our knowledge, been reported to colocalize in the same cells in the healthy adult brain. There is one report of NG2-positive OPCs being engulfed by CX_3_CR1-GFP-positive amoeboid microglia in the corpus callosum of developing mouse brains (Nemes-Baran et al., 2020). Furthermore, there are reports suggesting pericytes may differentiate into microglia in disease states and thereby begin expressing microglial markers (Özen et al., 2014; Sakuma et al., 2016). Conversely, others have reported expression of NG2 in microglia (Zhu et al., 2016; Huang et al., 2020). In our tissue however, a visual inspection of the rare dual-classified DsRed and GFP-positive cells revealed these were individual DsRed and GFP-positive cells with nuclei that were in close proximity (Fig. 3*E*,*F*), preventing the automated cell detection from separating the nuclei and consequently classifying them as the same cell. Although these dual classified cells were a rare occurrence, the inability of QuPath (and other automated cell detection programs) to accurately segregate close nuclei remains one of the limitations of automated cell counting. This limitation is best overcome by manual cell counting approaches, such as stereology.

We quantified microglia and pericytes in several brain regions, observing some significant differences between regions. Microglia are thought to account for ∼10% of the total number cells in the human brain and ∼5–10% in the mouse brain, although these numbers vary across brain regions (Lawson et al., 1990; von Bartheld et al., 2016). These numbers are consistent with our study where we found 4.51% of total cells were microglia, with the highest prevalence of GFP-positive microglia in the cortex. The precise percentage of cells that are microglia in mouse brains has rarely been quantified. Recently, Dos Santos and colleagues reported that microglia represent ∼6% of all cells in the mammalian cerebral cortical gray matter, after pooling data from 30 species (Dos Santos et al., 2020). For mice specifically, one previous study reported F4/80+ microglia accounted for ∼5–12% of the total number of cells in the mouse brain, depending on the region analyzed (Lawson et al., 1990). In particular, Lawson et al. (1990) found ∼5% of cells in the cerebral cortex were F4/80+ microglia, which compares favorably to our data from the cortex (5.54% of total cell detections). The small differences between our studies and others may be accounted for by the precise anatomic regions analyzed, our use of the CX_3_CR1 promoter to drive GFP expression in microglia, differences in tissue processing, quantification methodologies and the strain of mouse we used (C57/BL6).

The precise percentage of brain cells that are pericytes is more difficult to compare as the quantification of pericytes has not traditionally been included in most brain cell counting studies (von Bartheld et al., 2016). It is estimated that endothelial cells, which form blood vessels and on which pericytes reside, account for ∼30% of non-neuronal cells in the brain, and non-neuronal cells account for ∼50% of all brain cells (von Bartheld et al., 2016). Considering pericytes provide extensive coverage of endothelial cells (Berthiaume et al., 2018), and that there is an approximate ratio of one pericyte for every three endothelial cells in the brain (Pardridge, 1999), this equates to ∼5% of all cells in whole-brain sections that are possibly pericytes. This is consistent with our study where 3.71% of all cells were detected as pericytes, albeit with the caveat that a small proportion of the NG2-positive cells in our tissue are possibly OPCs (i.e., NG2 glia). In our study, the thalamus was found to have over twice the proportion of DsRed-positive cells compared with the other brain regions assessed. This may be because of the mouse thalamus having an increased vascular volume compared with other brain regions (Xiong et al., 2017), which could reflect the high amount of information that gets transmitted through the thalamus into other brain regions (Sherman and Guillery, 2002). Therefore, pericytes may play an active role in providing energy supply to this important brain region.

The relative number and spatial distribution of both microglia and pericytes can be drastically altered in disease states. Pericyte dysfunction and death are implicated in the pathogenesis of various brain diseases including stroke (Hall et al., 2014) and Alzheimer’s disease (Nortley et al., 2019), while microglia can readily migrate and alter their morphology and function in disease states (Bachiller et al., 2018). Therefore, the development of rapid and reliable tools like QuPath to quantify alterations in these cell populations will enhance our understanding of these cells in both health and disease.

Although our manual and automated cell counts were highly correlated, cell detection with QuPath is not yet perfect, as illustrated by the examples in Figure 3. For both GFP and DsRed fluorescent proteins, some false positives were detected because of high fluorescence from out-of-focus cells, cells adjacent to areas of high background, when a cell process was overlaying a DAPI-positive nucleus, or, in the case of DsRed, when a cell was present on a large vessel and therefore not representing a pericyte. Measures that could be used to mitigate these effects include using thinner brain sections, imaging in more than one plane and z-stacking, and improving classification to exclude large vessels and areas of high background. While false positives and negatives may limit the accuracy of automated cell counts, this may be an acceptable trade-off where it is impractical to manually count vast numbers of cells. However, we recommend that researchers using automated cell detection approaches, such as the one detailed here, compare the outcomes to an area that has been manually counted, because if these issues are not overcome, automated cell detection should not replace current gold standard manual cell counting techniques such as stereology.

Here, we have followed a simple cell classification workflow to demonstrate the potential of QuPath as a research tool. We largely avoided intersample and intrasample variability in fluorescence quality through our use of genetically-encoded fluorescent protein expression, allowing the use of fluorescence intensity thresholding for our analysis. However, variability of cell counts between individual brain slices was evident (Fig. 5), and additional optimization steps could be further employed to reduce this variability and improve the accuracy of automated cell detection. Potential approaches include the use of a second slice from each animal, an iteration of the algorithm with a second threshold, or using a particular area as a reference for sensitivity normalization. However, this will require additional computational time and human input to perform. Another parameter that could be considered includes the use of cell morphology to confirm a positive cell detection, particularly as pericytes and microglia have such strikingly different morphology. Alternatively, a center of mass approach for each nuclear detection to determine DsRed-positivity or GFP-positivity may prevent the impact of areas or sections with high cell density and overlapping nuclei and cell bodies, but this limits the area required for colocalization and impairs the ability to detect cells if the fluorescent signal lies outside of the center of the nucleus. More refined results may be possible using the machine learning algorithms that are built into QuPath. These trainable algorithms are likely to be particularly useful for creating classifiers capable of identifying cells positive for specific markers in tissue with varying degrees of immunofluorescent staining/imaging quality in different biological samples, for example differing levels of background artefacts such as age-related lipofuscin autofluorescence and large DsRed-positive vessels. In addition, QuPath interacts with ImageJ (among other packages), opening increased possibilities for analysis outside the simple methods shown here. Even within our workflow, QuPath generates more data than we have presented. For each detected cell, numerous other parameters are automatically measured including nuclear size and shape, and *XY* coordinates that can be used for further spatial analysis. Some of these analyses are already built into QuPath and others could be scripted as required or the data exported for analysis elsewhere. Further development of this methodology could include automating the analysis of tissue sections that have been imaged across multiple planes.

In conclusion, QuPath offers a user-friendly solution to whole-slide image analysis which will decrease reliance on down-sampling and region of interest analysis. Our novel scripts provide an automated workflow enabling the quick and efficient detection of both pericytes and microglia in the mouse brain. This pipeline enabled the detection of significant differences in microglial and pericyte cell numbers in different brain regions. The workflows we employed, and other functions within QuPath, make this a reliable automated image analysis tool for cell counting in fluorescently-labeled tissue that could lead to important new discoveries in both health and disease.

## Synthesis

Reviewing Editor: Francisca Bronfman, Universidad Andrés Bello Decisions are customarily a result of the Reviewing Editor and the peer reviewers coming together and discussing their recommendations until a consensus is reached. When revisions are invited, a fact-based synthesis statement explaining their decision and outlining what is needed to prepare a revision will be listed below. The following reviewer(s) agreed to reveal their identity: Oscar Lazo.

The technical concern of the reviewers has been resolved since robust statistical justifications have been provided for the aspects that were before insufficiently explained or methodologically obscure. Some minor points still remain: 1-please provide the complete curve of the counting of DAPI particles, the curves should converge into a certain value when increasing the threshold before dropping to -100% (or absolute zero). It is important to show that this convergence occurs; 2-in lines 346 and 458, it is mentioned “endogenous fluorophores” when referring to genetically encoded fluorescent proteins, please correct this statement"
