# Supplementary figures and images for: An Automated Approach to Improve the Quantification of Pericytes and Microglia in Whole Mouse Brain Sections
Source: eNeuro. 2021 Nov 3;8(6):ENEURO.0177-21.2021. doi: 10.1523/ENEURO.0177-21.2021 (PMC8570687; doi:10.1523/ENEURO.0177-21.2021)

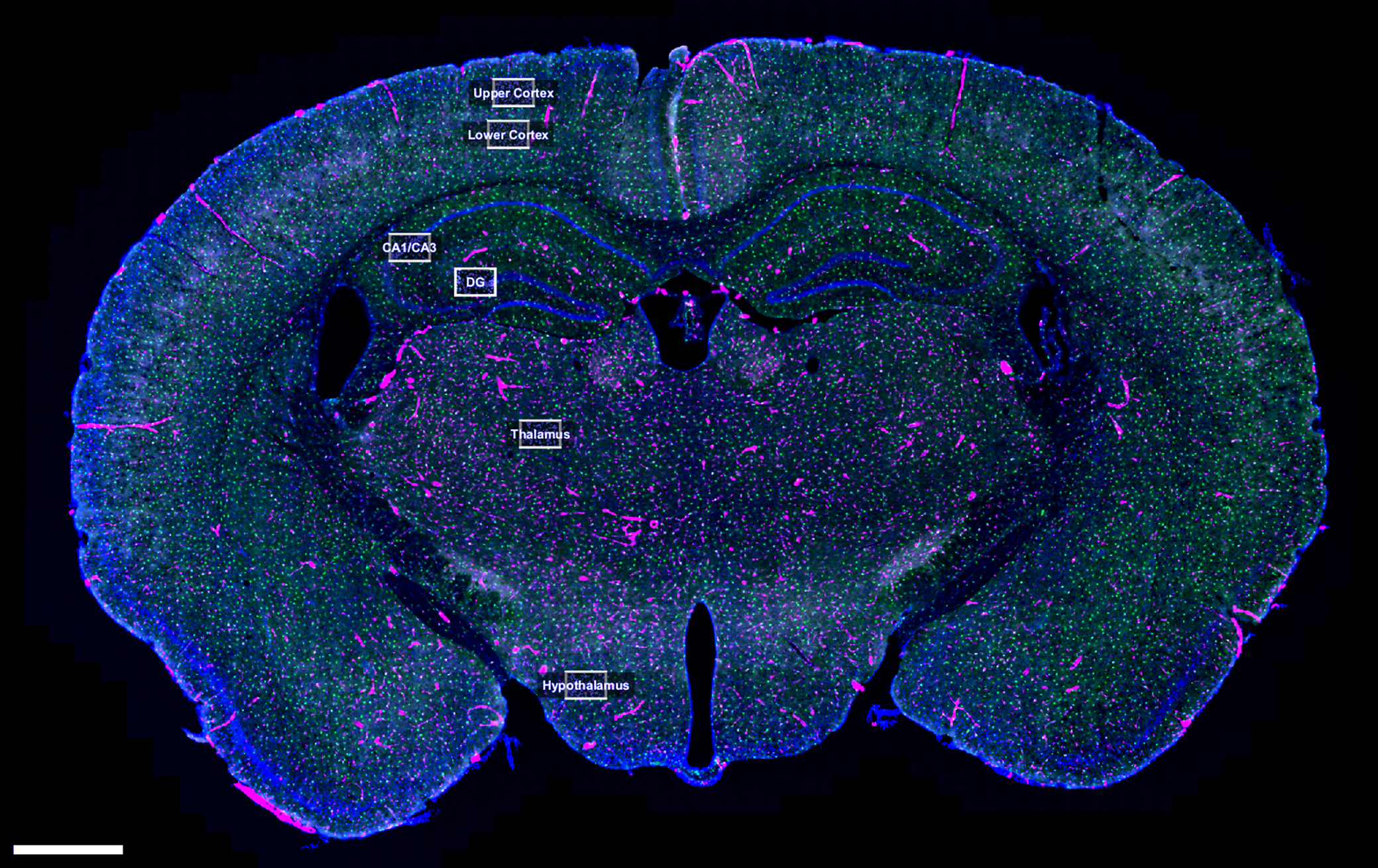

Supplement: Extended Data Figure 2-1 — Example of optimization annotations. We placed 300 × 200-μm regions of interest in the upper cortex (layers 1–3), lower cortex (layers 4–6), hippocampus (including dentate gyrus), hippocampus (including CA1/CA3 boundary), thalamus, and hypothalamus of each brain section for the purposes of manually counting cells. Scale bar: 800 μm. Download Figure 2-1, TIF file. [file enu-eN-MNT-0177-21-s02.tif]

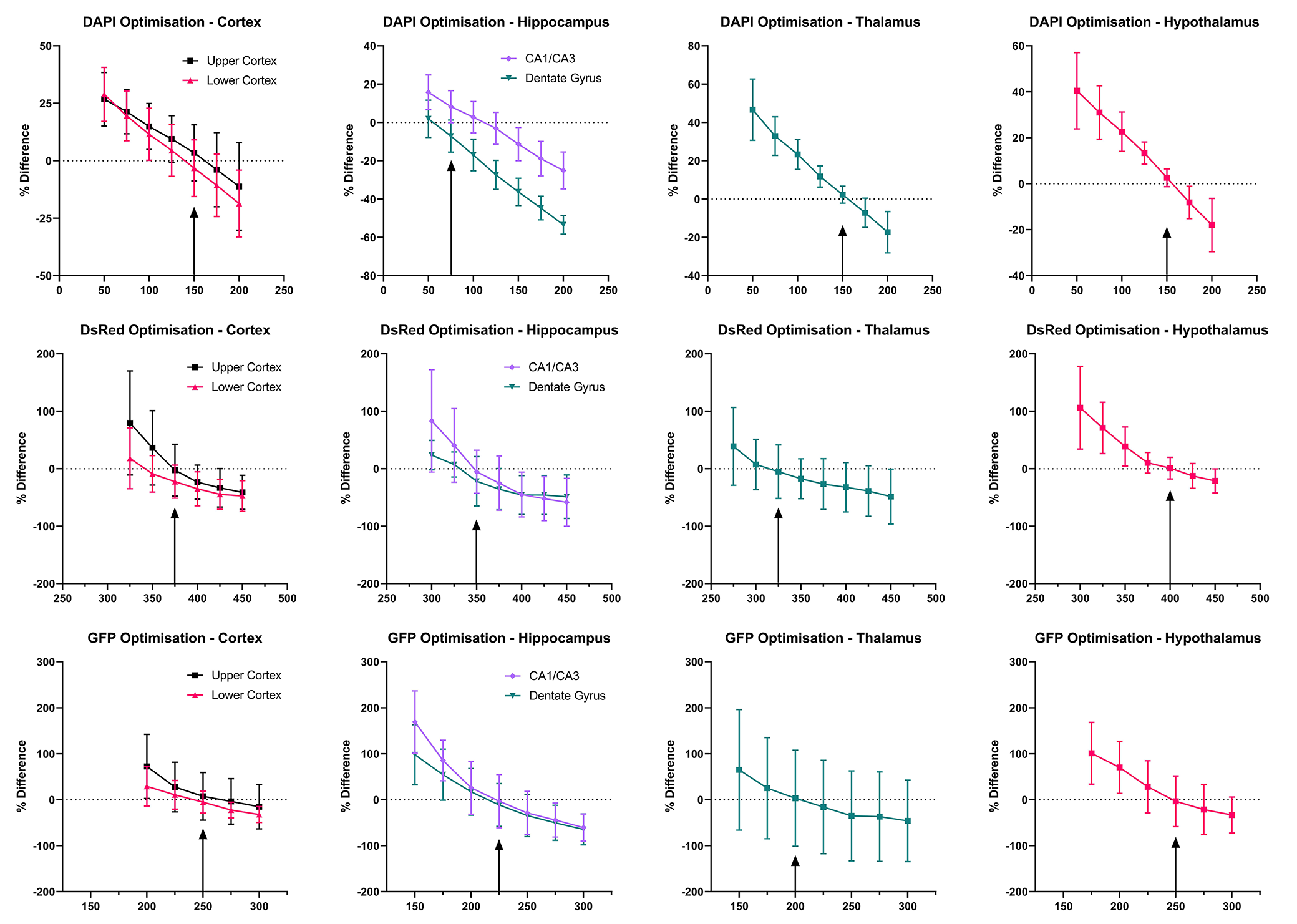

Supplement: Extended Data Figure 4-1 — Detailed optimization of cell detection and classification thresholds. Counts generated by QuPath’s cell detection/positive cell detection algorithm were compared to manual cell counts to generate a % difference (dotted line at 0%) with a range of intensity thresholds across six brain regions for DAPI, DsRed, GFP (n = 8, mean ± SD). Data for lower thresholds with large SDs have been excluded from the graphs in order to clearly visualize the optimum threshold for each region and channel (indicated with an arrow). Download Figure 4-1, TIF file. [file enu-eN-MNT-0177-21-s03.tif]

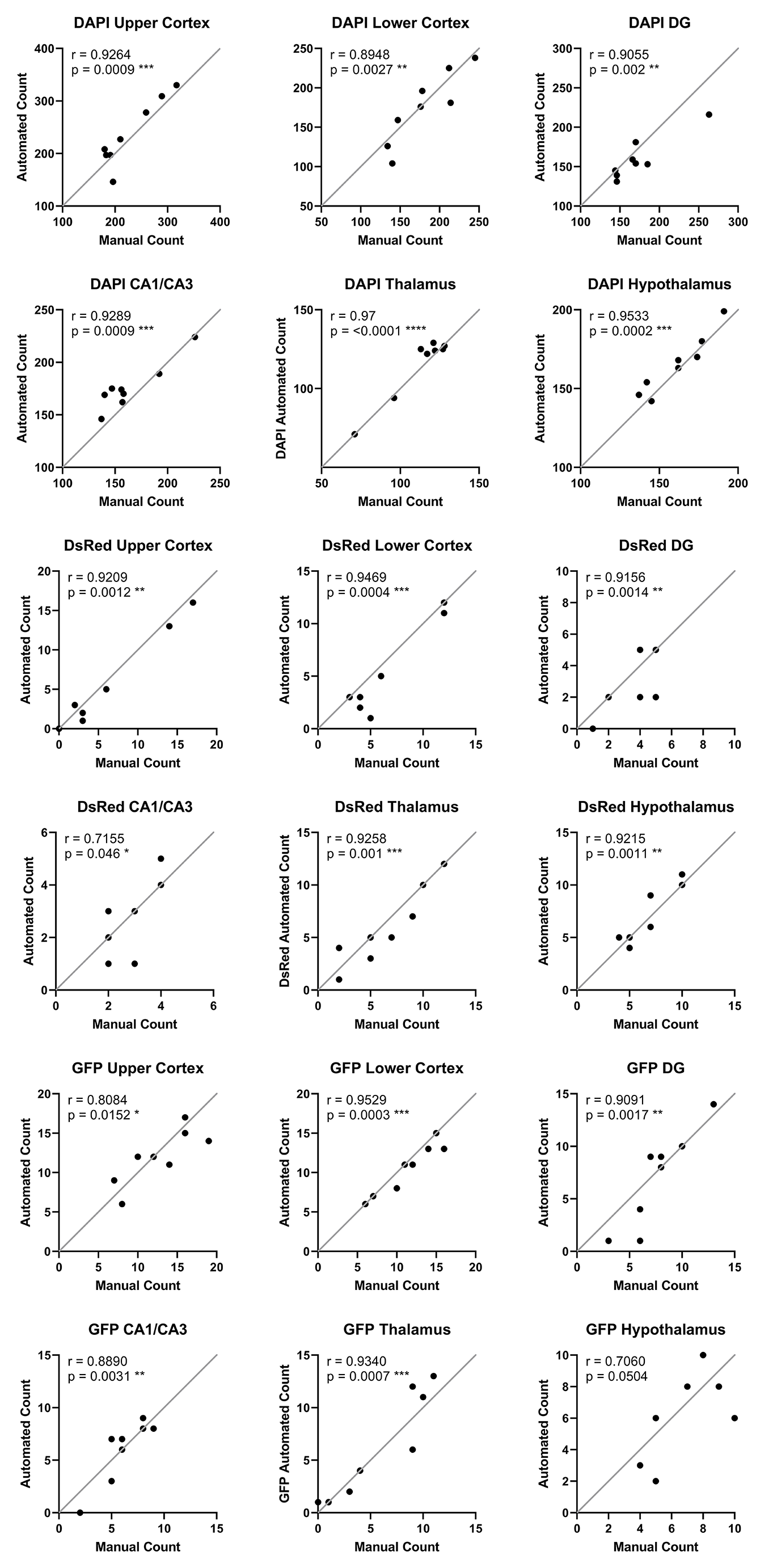

Supplement: Extended Data Figure 4-2 — Correlation of manual counts to automated counts at final optimized thresholds. For each optimized threshold, the Pearson’s correlation coefficient (r) between cells counted manually and automated counts by QuPath was calculated. Download Figure 4-2, TIF file. [file enu-eN-MNT-0177-21-s04.tif]

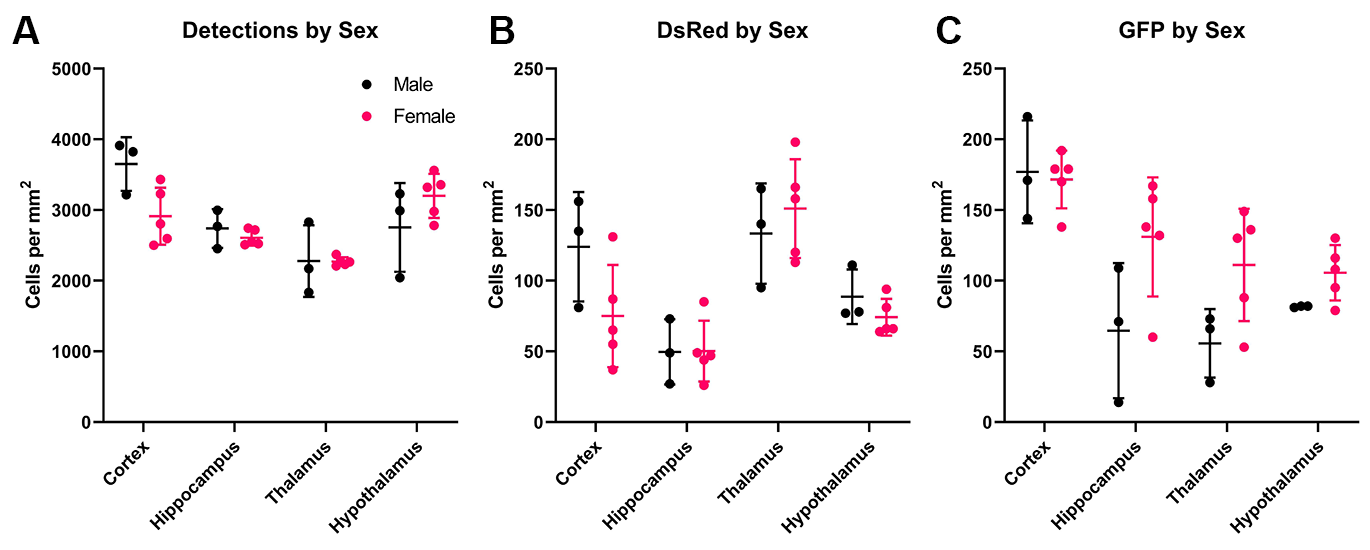

Supplement: Extended Data Figure 5-1 — Detection and classification of cells by sex. Total cells (A), DsRed-positive cells (B), and GFP-positive cells (C) detected per mm2 tissue area. No effect of sex was found by two-way ANOVA. Download Figure 5-1, TIF file. [file enu-eN-MNT-0177-21-s05.tif]
